# Circulating Bacterial DNA: A New Paradigm for Cancer Diagnostics

**DOI:** 10.3389/fmed.2022.831096

**Published:** 2022-04-04

**Authors:** Tamara Glyn, Rachel Purcell

**Affiliations:** Department of Surgery, University of Otago, Christchurch, New Zealand

**Keywords:** cancer, diagnostic test, bacterial DNA, microbiome, liquid biopsy, cell-free DNA

## Abstract

Cell-free DNA applications for screening, diagnosis and treatment monitoring are increasingly being developed for a range of different cancers. While most of these applications investigate circulating tumor DNA (ctDNA) or methylation profiles of ctDNA, circulating bacterial DNA (cbDNA) has also been detected in plasma and serum samples from cancer patients. Recent publications have the detection of cbDNA in studies of breast, gastric, colorectal, hepatocellular and ovarian cancers. In several cases, distinction between patients and healthy controls was possible, based on cbDNA profiles, in addition to potential prognostic value. A large pan-cancer study demonstrated the feasibility of cbDNA to distinguish between four types of cancer and healthy controls, even in patients with early-stage disease. While improvements in, and standardization of laboratory and bioinformatics analyses are needed, and the clinical relevance of cbDNA yet to be ascertained for each cancer type, cbDNA analysis presents an exciting prospect for future liquid biopsy screening and diagnostics in cancer.

The concept of a liquid biopsy for monitoring cancer progression or treatment response has become increasingly popular, largely due to technical improvements in the ability to measure and analyze small amounts of cell-free (cf) DNA and RNA in plasma, and is in clinical use in some diagnostic centers in the US and Europe. The advantages of a liquid biopsy approach include the minimal invasiveness of a blood test compared to tumor biopsy, and repeatability of testing over time (1). Circulating tumor DNA (ctDNA) containing tumor-specific mutations can be used to predict outcome and monitor response to treatment in several types of solid tumors, including melanoma and lung cancer, with many uses currently in clinical trial. However, a major drawback of current use is that a common cancer mutation must be identified in the primary tumor, and then this mutation must also be detectable in the tiny amount of ctDNA, a veritable “needle in a haystack.”

Cell-free DNA acts like a genetic reservoir that carries genetic information from all cells within the body (2), including healthy and diseased cells and microbes (3). Applications of cfDNA sequencing in oncology have been increasingly explored (4, 5), however as of yet, little attention has been paid to the identification and characterization of circulating bacterial DNA (cbDNA) in the oncologic context. To date, identification of microbes in the circulatory system has mainly been applied to infectious disease and sepsis, where sequencing of cfDNA has improved detection of micro-organisms that are difficult to culture, and has augmented traditional culture techniques (6, 7). Emerging research in the field of cfDNA has identified highly divergent cbDNA in a variety of non-communicable diseases, including liver (8), metabolic (9), autoimmune (10), and cardiovascular disease (11, 12). Although the source, route of access and significance of this cbDNA in disease states has yet to be fully elucidated (3), several studies have identified DNA from common gut commensal bacteria in patient plasma samples.

Strong associations between alterations in the gut microbiome (dysbiosis) and numerous non-infectious diseases, including those mentioned previously, have been extensively reported in the literature. The role of the microbiome in cancer is no exception, with well-established links to disease progression and response to therapy in a wide variety of tumor types (13–15). Microbiome-based diagnostic testing and interventions are currently being investigated, and interest in the potential role of cbDNA in cancer has also increased in recent years. While the microbiome includes bacteria, viruses, fungi and archea, the bacterial component is the most well-studied, and this also holds true for studies carried out examining circulating DNA; hence, we focus on cbDNA in this review (Figure 1; Table 1).

**Figure 1:**
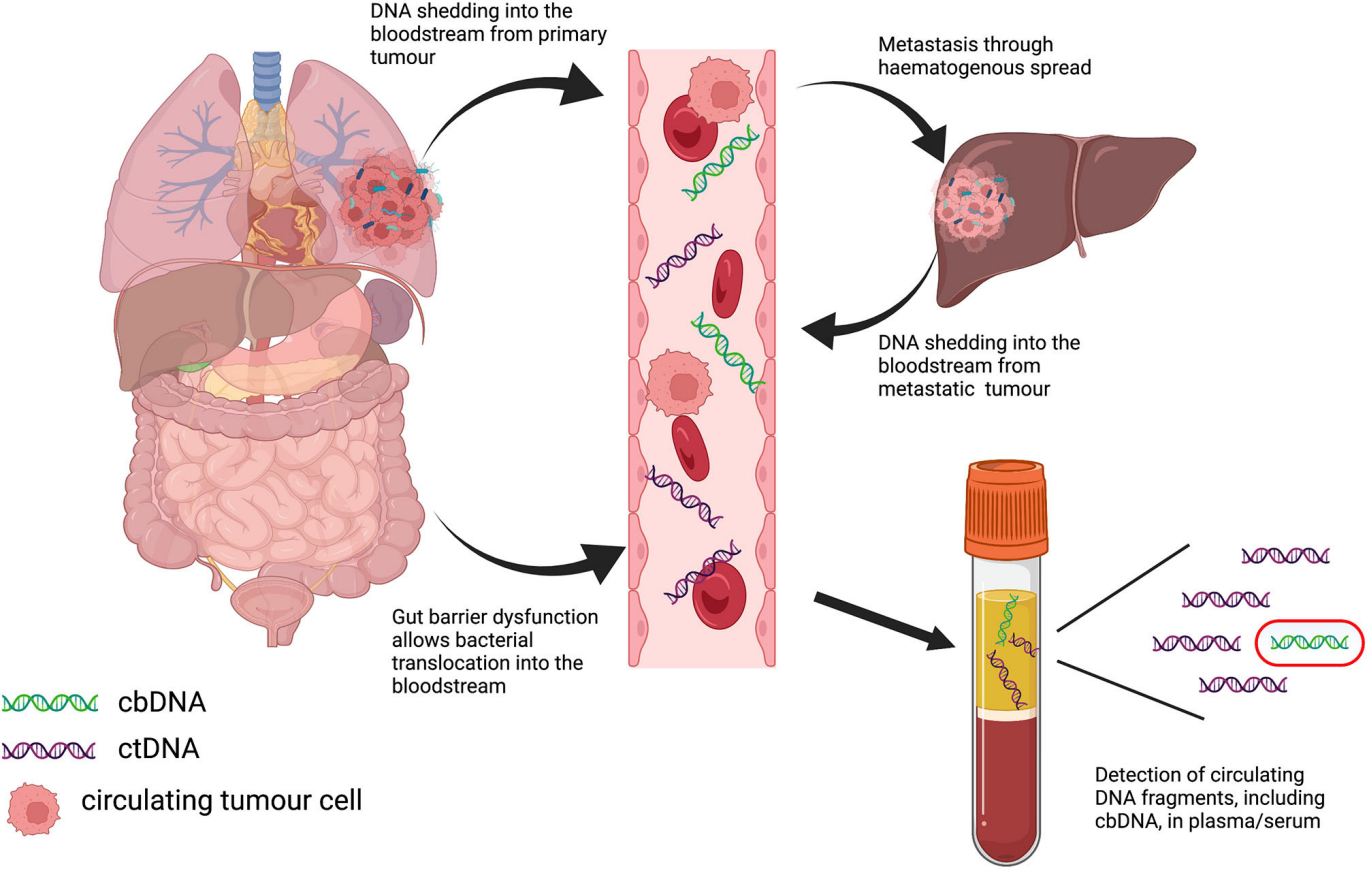
Graphical depiction of potential routes of translocation of bacterial DNA into the bloodstream in cancer, and subsequent detection in plasma samples.

**Table 1:** Published studies of circulating bacterial DNA in cancer.

**Cancer type**	**Methodology**	**Main findings**	**Study**
Breast	Metagenomics analysis of plasma DNA	cbDNA identified as a potential prognostic indicator	Huang et al. (16)
Gastric	16S rRNA sequencing of serum samples	Lower alpha diversity in cancer patients compared to controls; specific taxa correlate with clinical indices	Dong et al. (17)
Hepatocellular	16S rRNA sequencing of serum samples	Lower alpha diversity in cancer patients compared to controls; Differentially abundant taxa between cancer and controls; Development of 5-microbial gene marker panel	Cho et al. (18)
Ovarian	Metagenomic analysis of bacterial DNA derived from extra-cellular vesicles from serum samples	Different metagenomic profiles between cancer and controls. Acinetobacter common to cancer samples	Kim et al. (19)
Colorectal	Metagenomics analysis of plasma DNA	Slightly lower diversity in cancer samples; cbDNA mainly from gut-associated species; 28-species model could distinguish cancer from controls.	Xiao et al. (21)
	PCR amplification of specific microbial targets (*16S* gene, *E. coli, B. fragilis, C. albicans*) from whole blood samples	Higher detection of all fragments, except *E. coli*, in cancer samples; detection of microbial fragments associated with metastasis	Messaritakis et al. (22)
	PCR amplification of specific microbial targets (*16S* gene, *E. coli, B. fragilis, C. albicans*) from whole blood samples	Association between detection of microbial fragments and circulating tumor cells	Koulouridi et al. (23)
Multiple cancer types	Whole genome sequencing of whole blood samples; metagenomic analysis of plasma samples	Circulating microbial DNA profiles can distinguish between multiple types of cancer, including low-grade tumors; similar discrimination seen in plasma analysis	Poore et al. (24)

The first reported study of cbDNA in cancer reported its potential as a prognostic indicator in a study of a small number of women with early-onset breast cancer (16). The authors reported that the cbDNA from both patients and healthy controls was predominantly bacterial in origin and limited to a small number of genera. A key finding of this study was that microbial taxa were more diverse in patients compared to controls. A patient with a similar cbDNA profile to that of healthy controls was disease-free after more than 10 years of follow-up. In contrast, patients with more diverse taxa had short disease-free survival, suggesting the potential for cbDNA as a prognostic indicator.

While the aforementioned study used a metagenomics approach to analyse cbDNA in cancer patients, several other studies have looked at the circulating microbiome using an amplicon sequencing method, namely 16S rRNA sequencing, or DNA detection of specific bacterial species of interest using PCR. 16S rRNA is commonly used in microbiome studies to define the bacterial taxonomic composition to the genus level and analyse diversity between samples or environmental conditions. Dong et al. used 16S rRNA sequencing of plasma samples to compare cbDNA from patients with gastric cancer, atypical hyperplasia, chronic gastritis and healthy controls (17). Alpha diversity was significantly lower in gastric cancer patients compared to chronic gastritis patients or healthy controls, and genus-level analysis showed similar profiles between gastric cancer and atypical hyperplasia. Significant correlations were also reported between circulating DNA from specific bacterial genera and clinical indices, such as lymphatic metastases and tumor. A similar approach used 16S rRNA sequencing data from serum samples from patients with hepatocellular cancer, cirrhosis and healthy controls (18). In this study, microbial diversity was also reduced in cancer patients compared to non-cancer patients and controls, with differences in relative abundance of taxa between patients with hepatocellular cancer and controls. A five microbial gene marker model, based on these initial findings, could differentiate cancer patients from controls with >80% accuracy.

Extracellular vesicles (EV) have also been shown to contain microbial DNA arising from the gut microbiome and this phenomena has been exploited to investigate bacterial EV-derived DNA in the circulation of patients with ovarian cancer (19). Comparison of 16S rRNA sequencing data uncovered a significant difference in the abundance of *Acinetobacter* between ovarian cancer patients compared to patients with benign ovarian tumors.

Research into the contribution of the gut microbiome to the development and progression of colorectal cancer (CRC) is an area of intense interest, given the proximity of colorectal tumors to the gut microbiome. Sequencing studies have highlighted the taxonomic differences in the microbiota of CRC patients compared to healthy controls and in tumor tissue compared to matched normal, while functional studies have identified potential mechanisms of action for certain bacterial species, such as *Bacteroides fragilis* and *Fusobacterium nucleatum* in colorectal carcinogenesis. Increased concentrations of cfDNA have been reported to correspond to higher stage in CRC (20), suggesting that tumor DNA is present in the circulation either as part of the haematogenous spread of the primary tumor or due to increased shedding of tumor DNA into the circulatory system from the metastatic site, which is predominantly the liver. Xiao et al. reported the use of metagenomic analysis of plasma samples to investigate the utility of cbDNA as a diagnostic marker in CRC (21). The study compared whole genome sequencing data from plasma samples of CRC and colorectal adenoma patients, and healthy volunteers and found that cbDNA profiles could distinguish between the three patient groups. While the cohorts were small, a classifier model based on 28 species successfully distinguished between the three groups using a separate validation cohort. Two separate studies from a Greek research group have reported the utility of measuring specific bacterial species in the blood, namely *E. coli, B. fragilis* and *C. albicans*, for prognostic (22) and predictive (23) purposes. They found that detection of DNA from these microbes using PCR was associated with metastatic disease and shorter survival, whereas the association with circulating tumor cells and response to therapy was less clear.

The most comprehensive pan-cancer study to date was recently published by Poore et al., and described how whole-genome sequencing data from blood and tissue microbiomes can discriminate between 33 different cancer types using data from The Cancer Genome Atlas (TCGA) (24). Using plasma samples from a separate cohort, the authors were also able to distinguish between healthy controls and four types of cancer using cbDNA signatures. A potentially transformational finding from this study was that the cbDNA signatures remained predictive of cancer type, even in Stage I and II cancers, and in cancers lacking any genomic alterations. This is an important point, as current circulating tumor DNA (ctDNA) analysis relies on detecting common genomic alterations that are present in the primary tumor, and implies that cbDNA may be a more sensitive and widely applicable cancer biomarker.

From the currently available literature, there is accumulating evidence that cbDNA may have diagnostic and/or prognostic value in cancer. However, the source of cbDNA in cancer, whether it be from direct shedding into the bloodstream from primary or metastatic tumors, intestinal barrier dysfunction (leaky gut), or other unidentified mechanisms, remains unclear, and future research should address the source and clinical importance of cbDNA in any given cancer type. As with microbiome analysis of other sample types, such as tumor tissue and stool samples, the analytical method (metagenomic, 16S amplicon-based or PCR) will greatly impact the findings and make comparisons difficult. Different sequencing platforms and bioinformatics tools will also influence the outcome of cbDNA studies, and benchmarking should be carried out to ascertain the best practice for cbDNA analysis. Consistent with other low-biomass microbiome studies, contamination is also an area of concern, whether physically introduced through analytical reagents and kits, or due to errors and inconsistencies in microbial DNA databases used for identification, and future efforts must include robust controls to mitigate these effects. The development of cbDNA as a diagnostic tool for cancer faces many challenges and is very early in its development, particularly compared to ctDNA, which has already shown to have clinical utility. However, future large-cohort studies with robust sequencing and analytical methodologies may identify potential clinical applications, particularly in the area of screening and early detection for a wide range of malignancies.

## Author Contributions

Both authors listed have made a substantial, direct, and intellectual contribution to the work and approved it for publication.

## Conflict of Interest

The authors declare that the research was conducted in the absence of any commercial or financial relationships that could be construed as a potential conflict of interest.

## Publisher's Note

All claims expressed in this article are solely those of the authors and do not necessarily represent those of their affiliated organizations, or those of the publisher, the editors and the reviewers. Any product that may be evaluated in this article, or claim that may be made by its manufacturer, is not guaranteed or endorsed by the publisher.
